# X-ray diffraction and electron microscopy data for amyloid formation of Aβ40 and Aβ42

**DOI:** 10.1016/j.dib.2016.05.020

**Published:** 2016-05-20

**Authors:** Olga M. Selivanova, Elizaveta I. Grigorashvili, Mariya Yu. Suvorina, Ulyana F. Dzhus, Alexey D. Nikulin, Victor V. Marchenkov, Alexey K. Surin, Oxana V. Galzitskaya

**Affiliations:** Institute of Protein Research, Russian Academy of Sciences, 142290 Pushchino, Moscow Region, Russia

## Abstract

The data presented in this article are related to the research article entitled “One of the possible mechanisms of amyloid fibrils formation based on the sizes of primary and secondary folding nuclei of Aβ40 and Aβ42” (Dovidchenko et al., 2016) [1]. Aβ peptide is one of the most intensively studied amyloidogenic peptides. Despite the huge number of articles devoted to studying different fragments of Aβ peptide there are only several papers with correct kinetics data, also there are a few papers with X-ray data, especially for Aβ42. Our data present X-ray diffraction patterns both for Aβ40 and Aβ42 as well for Tris–HCl and wax. Moreover, our data provide kinetics of amyloid formation by recombinant Аβ40 and synthetic Аβ42 peptides by using electron microscopy.

**Specifications Table**

**Table:** 

Subject area	*Biophysics*
More specific subject area	*Amyloid formation*
Type of data	*Table, Figures*
How data was acquired	*JEM-1200EX transmission electron microscope at the accelerating voltage of* 80 kV*, Microstar X-ray generator with HELIOX optics*
Data format	*Analyzed*
Experimental factors	*Samples were incubated to obtain amyloid fibrils*
Experimental features	*Temperature of incubation* 37 °C*, pH* 7.5, *Tris–HCl buffer, dissolutiong in DMSO*
Data source location	*Institute of Protein Research, Russian Academy of Sciences,* 142290 *Pushchino, Moscow Region, Russian Federation*
Data accessibility	*Data is within this article*

**Value of the data**

• X-ray diffraction patterns of Tris–HCl and wax are important for scientists because they give additional diffraction patterns resulting in difficult interpretation of data.
• Aβ peptide exhibits polymorphism. The morphologies of our samples may be interesting and useful in terms of collecting different examples of polymorphic fibrils and comparing them with the ones obtained by the other researchers.
• These data are valuable to researchers interested in studying amyloid formation of proteins and peptides.

## 1 Data

**Fig. 1:**
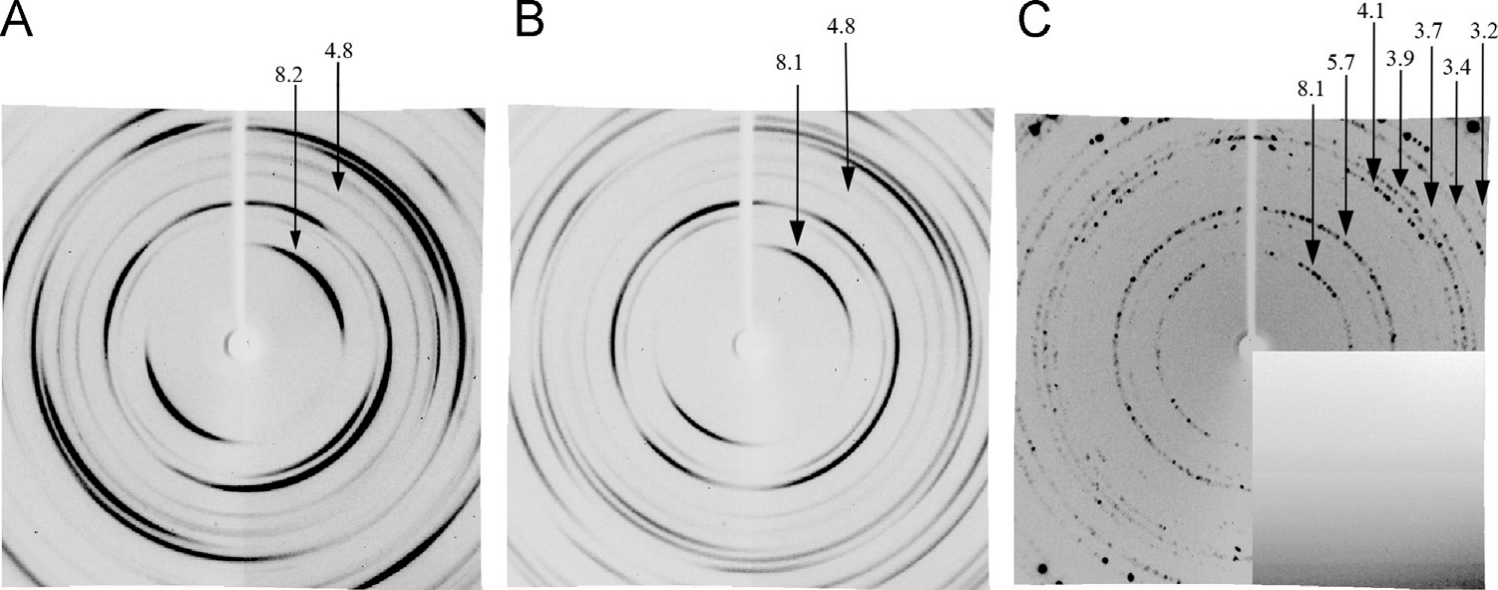
X-ray diffraction patterns of synthetic (Sigma) Aβ peptide fibrils: (A) Aβ40 peptide; (B) Aβ42 peptide; (C) 0.5 M Tris–HCl (pH 7.5).

**Fig. 2:**
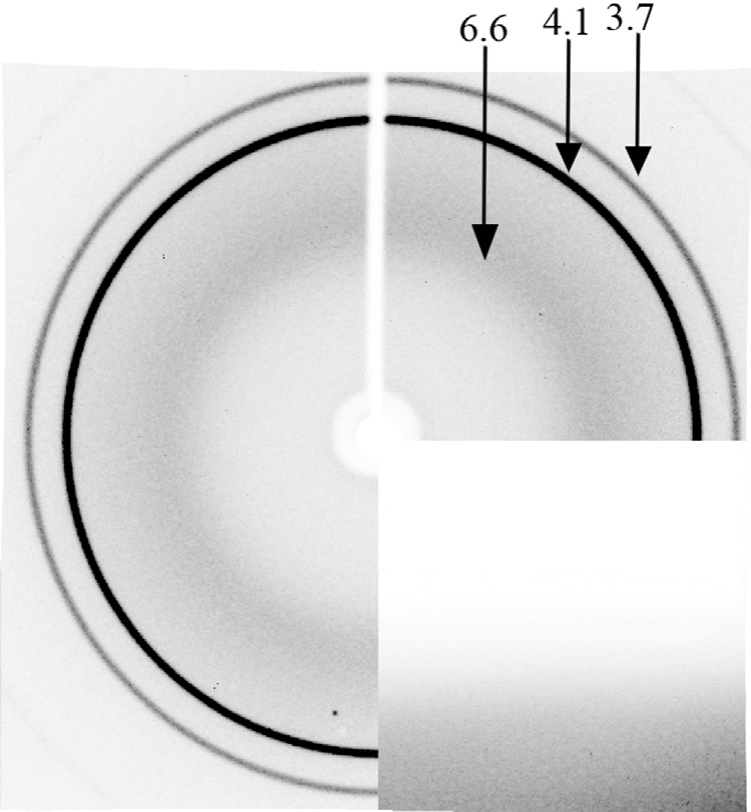
X-ray diffraction pattern of wax.

X-ray diffraction patterns of synthetic and recombinant Aβ40 and synthetic Aβ42 fibrils (Figs. 1 and 2, Table 1).

Kinetics of amyloid formation by recombinant Аβ40 peptide and synthetic Аβ42 peptide by using electron microscopy (Tables 2 and 3).

## 2 Experimental design, materials and methods

### 2.1 X-ray diffraction analysis

The recombinant Aβ40 and Aβ42 and synthetic Aβ42 (Sigma) peptides in 50 mM Tris–HCl (pH 7.0–7.2) for X-ray diffraction analysis were prepared after 7–14-day incubation at 37 °C [1]. The samples were concentrated down to 5–10 mg/ml at room temperature using an Eppendorf 5301 vacuum concentrator. Then the preparation droplets (~5 μL) were placed within the space (about 1.5 mm) between the ends of glass tubes (about 1 mm in diameter) coated with wax. After drying for 24 h, rod specimens 1–1.5 mm long and about 0.1 mm in diameter were obtained.

The fiber diffraction images were collected using a Microstar X-ray generator with HELIOX optics, equipped with a Platinun135 CCD detector (X8 Proteum system, Bruker AXS) at the Institute of Protein Research, RAS, Pushchino. Cu Kα radiation (*λ*=1.54 A) was used. The samples were positioned at the right angle to the X-ray beam using a 4-axis kappa goniometer. With such technique of specimen concentration, the Tris–HCl concentration can reach 1 M that interferes greatly with the interpretation of the obtained X-ray diffraction patterns. Fig. 1 shows the data evidencing that Aβ40 and Aβ42 synthetic peptides and Aβ40 recombinant peptide have reflections (see Table 1), coinciding with reflections from 0.5 M Tris–HCl (pH 7.5), in addition to the characteristic reflection for the cross-β structure (4.8 Ǻ and 8.1 Ǻ). One should take notice of the method of preparation of specimens for X-ray diffraction analysis and, if a sufficient amount of Aβ peptides is available, avoid its strong concentration as in our case. Researchers should also pay attention to the length of the preparation (dried rod) ready for X-ray analysis. It should be no less than 0.5 mm, otherwise X-ray diffraction can be obtained not of the preparation itself, but of wax (Fig. 2).

### 2.2 Electron microscopy

All the samples were initially dissolved in DMSO (the final concentration 5%), then the buffer (50 mM Tris–HCl, pH 7.5) was added. Prior to staining, the concentration of the samples was adjusted to 0.1 mg/ml. A copper grid (400 mesh) coated with a formvar film (0.2%) was mounted on a sample drop (10 µl). After 10 min absorption, the grid with the preparation was negatively stained for 1.5–2.0 min with 1% (weight/volume) aqueous solution of uranyl acetate. The excess of the staining agent was removed with filter paper. The preparations were analyzed using a JEM-1200 EX transmission electron microscope at the accelerating voltage of 80 kV. Images were recorded on the Kodak electron image film (SO-163) at nominal magnification of 40,000–60,000.

## Figures and Tables

**Table 1:** Comparison of X-ray diffraction patterns of amyloid fibrils of synthetic (Sigma) preparations Aβ40 and Aβ42 (concentrated from 0.05 M Tris–HCl, pH 7.5) and the preparation of 0.5 M Tris–HCl (pH 7.5). Reflections characteristic of cross-β structure are given in bold type.

Preparation	Reflections (Ǻ) of synthetic (Sigma) Aβ40 and Aβ42 peptides, recombinant Aβ40, and preparation of 0.5 M Tris–HCl (pH 7.5)									
Аβ1-40 Sigma		3.4	3.7	3.9	4.1	4.4	**4.9**	5.7	6.3	**8.1**
Аβ1-42 Sigma	3.2	3.4	3.7	3.9	4.1	4.4	**4.8**	5.7	6.3	**8.1**
Аβ1-40 recomb.	3.2	3.4	3.7	3.9	4.1	4.4	**4.8**	5.7	6.3	**8.1**
0.5 M Tris–HCl, pH 7.5	3.2	3.4	3.7	3.9	4.1			5.7		**8.1**

**Table 2:** Kinetics of amyloid formation by recombinant Аβ40 peptide (50 mM Tris–HCl, pH 7.5, 25 °С, 5% DMSO, *С*=0.2 mg/ml).

**Table 3:** Kinetics of amyloid formation by synthetic Аβ42 peptide (Sigma, 50 mM Tris–HCl, pH 7.5, 37 °С, 5% DMSO, *С*=0.1 mg/ml).
