# Supplementary figures and images for: X-ray diffraction and electron microscopy data for amyloid formation of Aβ40 and Aβ42
Source: Data Brief. 2016 May 20;8:108–13. doi: 10.1016/j.dib.2016.05.020 (PMC4889875; doi:10.1016/j.dib.2016.05.020)

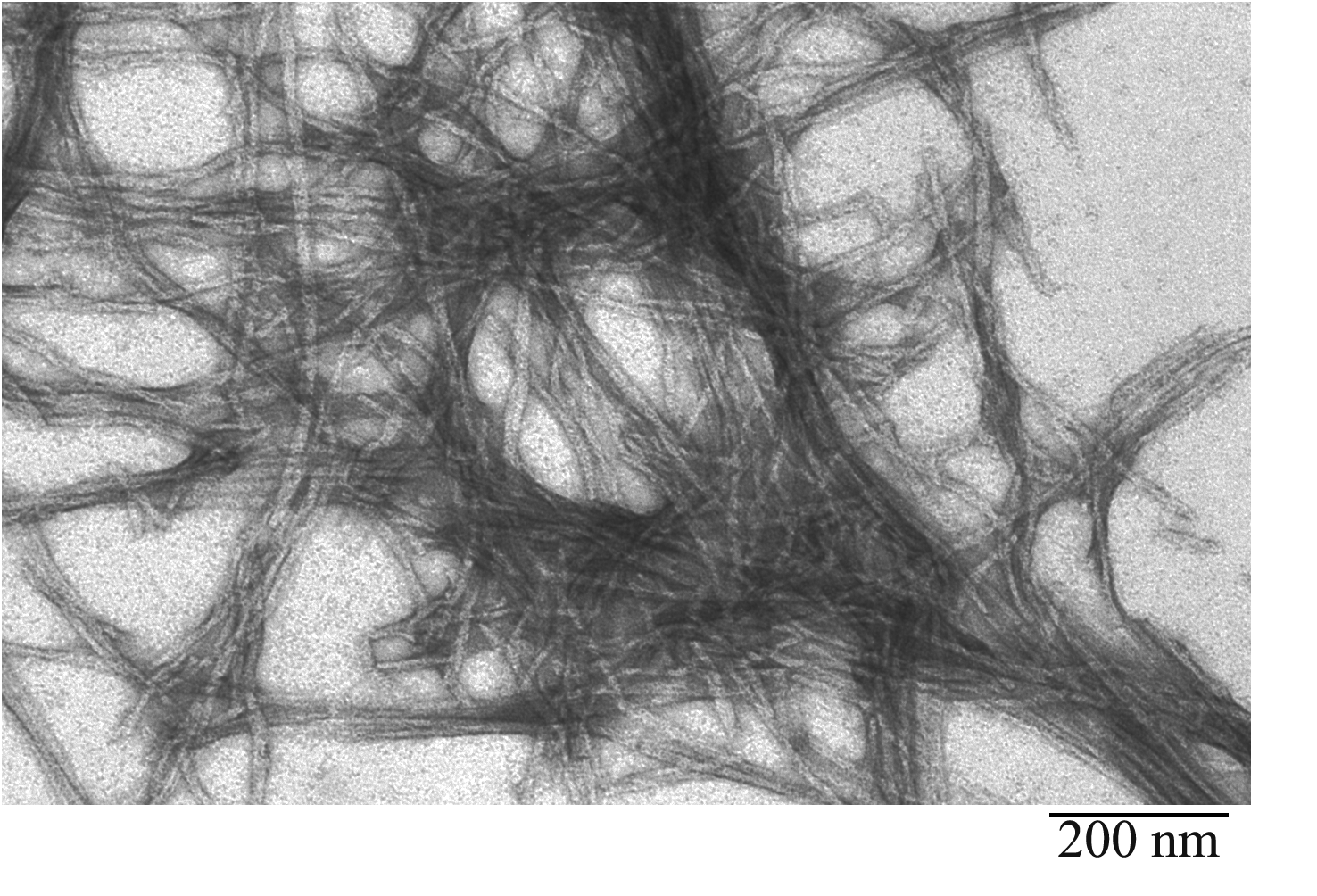

Supplement: Supplementary file 2 — Supplementary material [file mmc2.zip › RecAb40_55 h_Sup.tif]

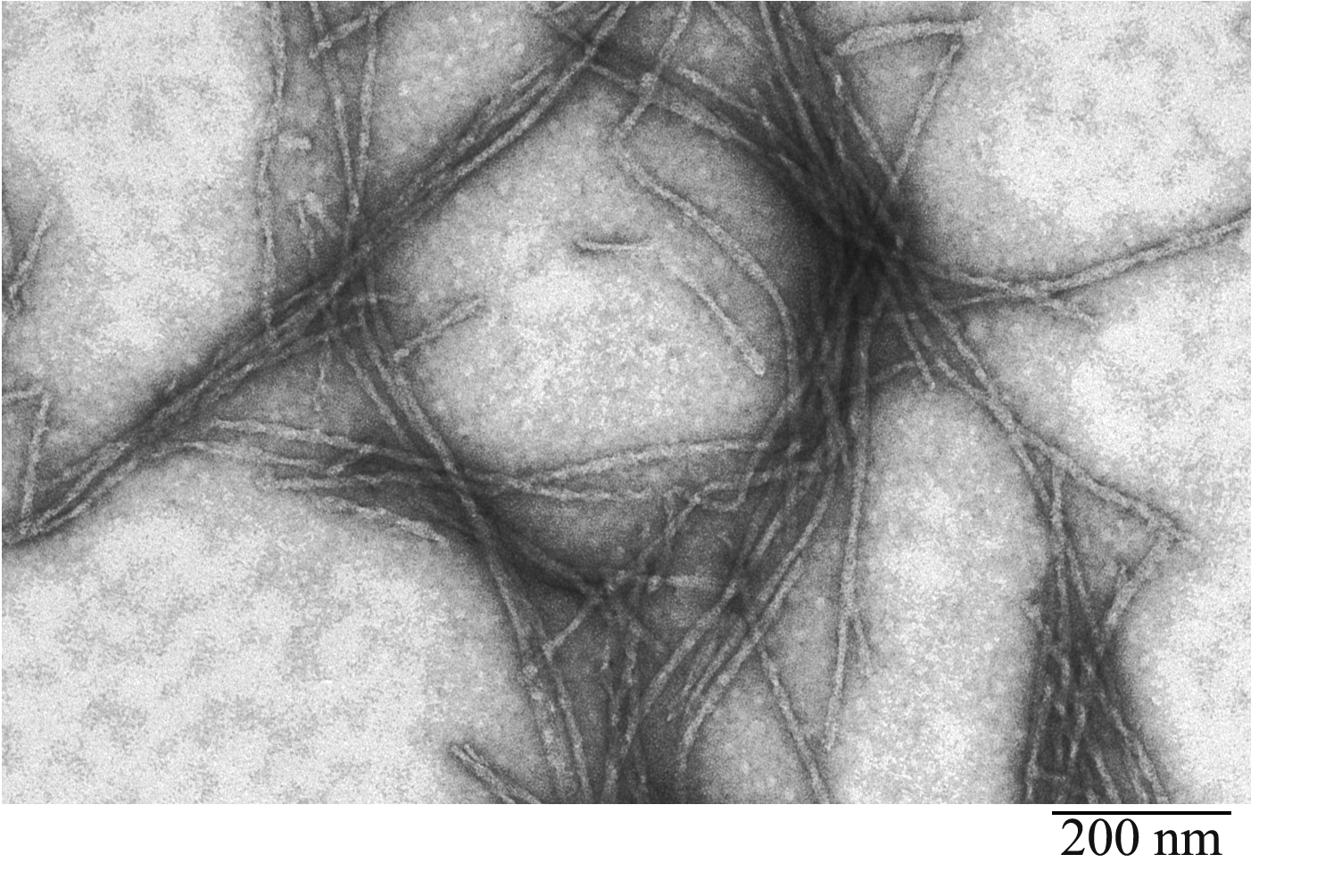

Supplement: Supplementary file 2 — Supplementary material [file mmc2.zip › RecAb40_27 h_Sup.tif]

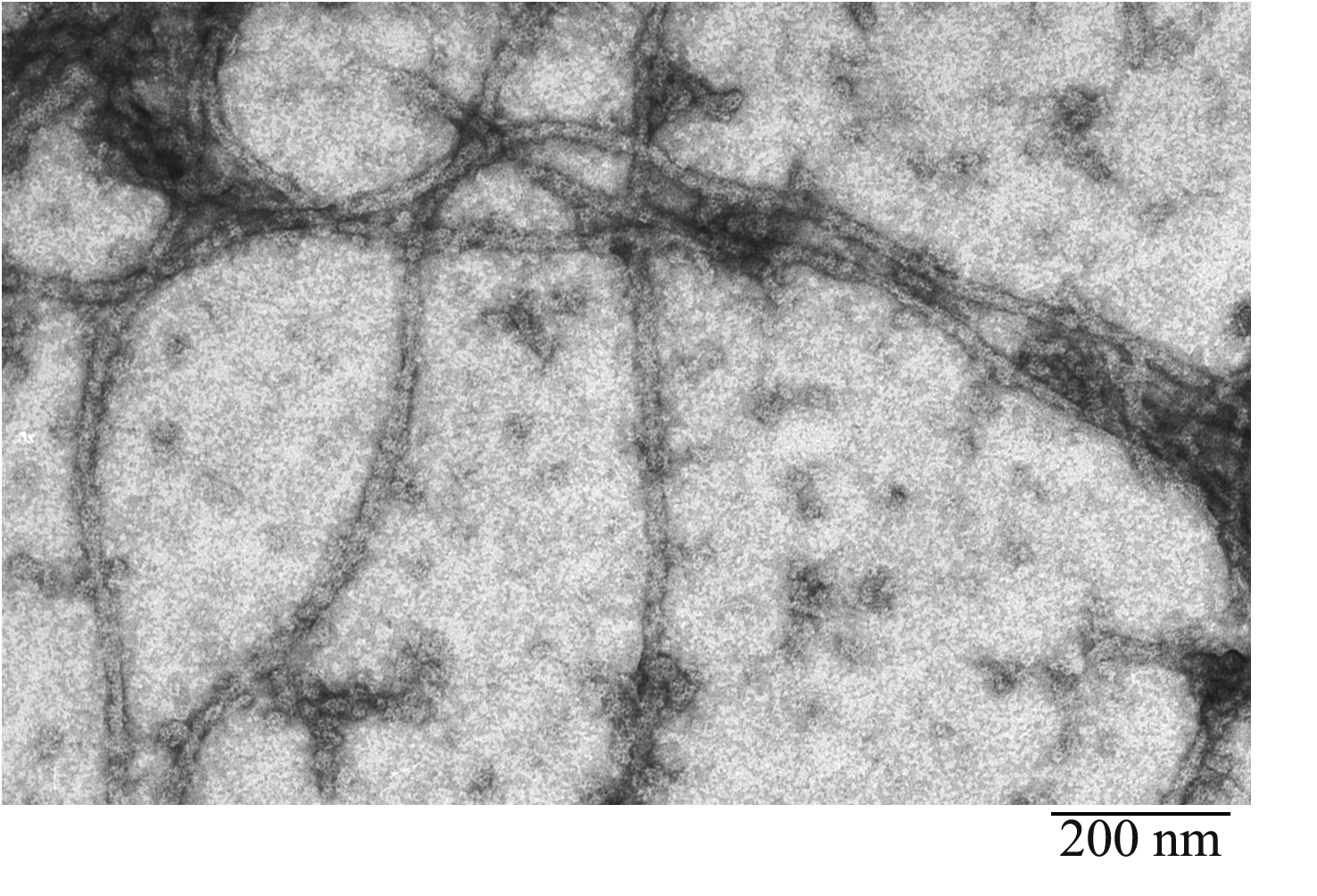

Supplement: Supplementary file 2 — Supplementary material [file mmc2.zip › RecAb40_8 h_Sup.tif]

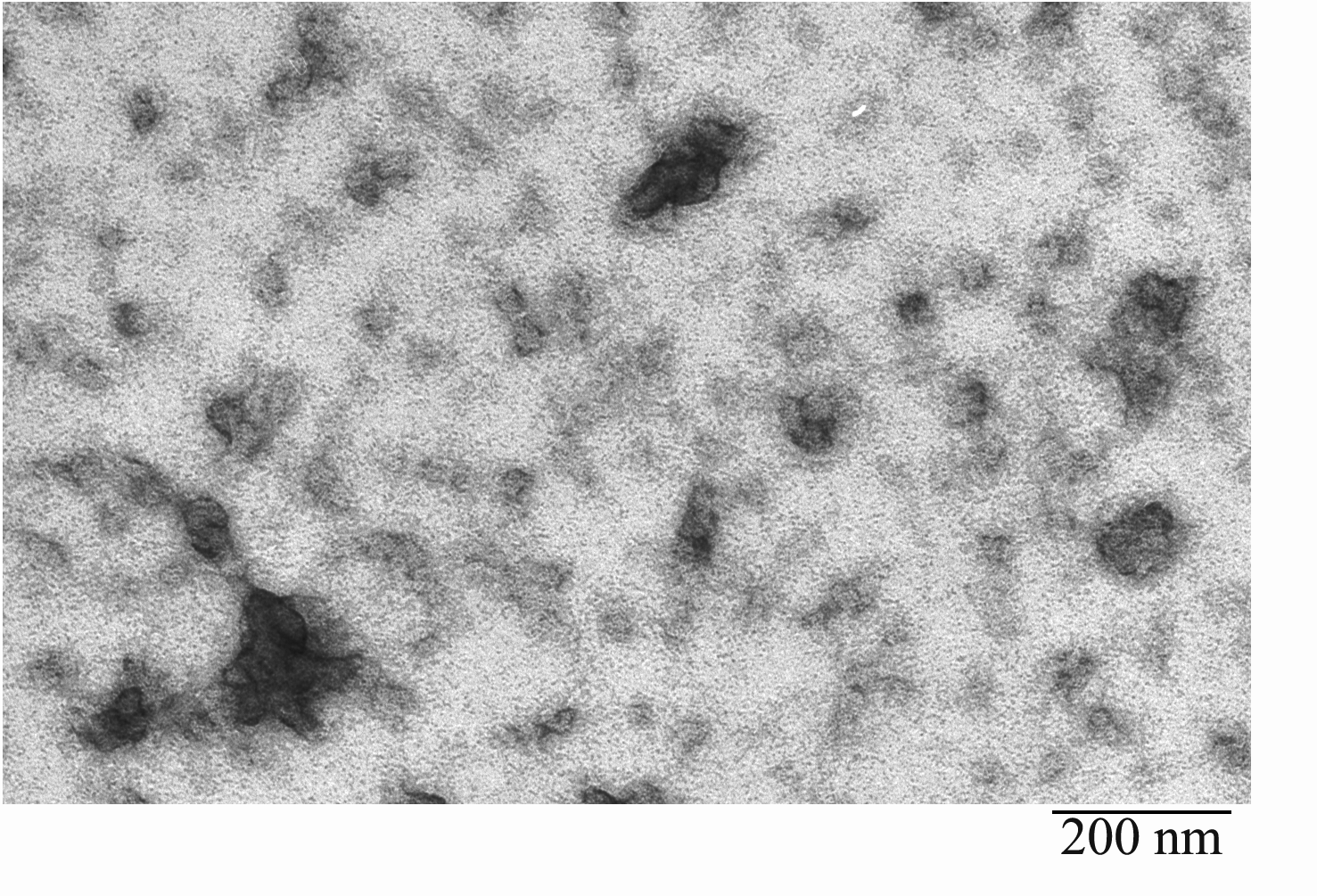

Supplement: Supplementary file 2 — Supplementary material [file mmc2.zip › RecAb40_0 h_Sup.tif]

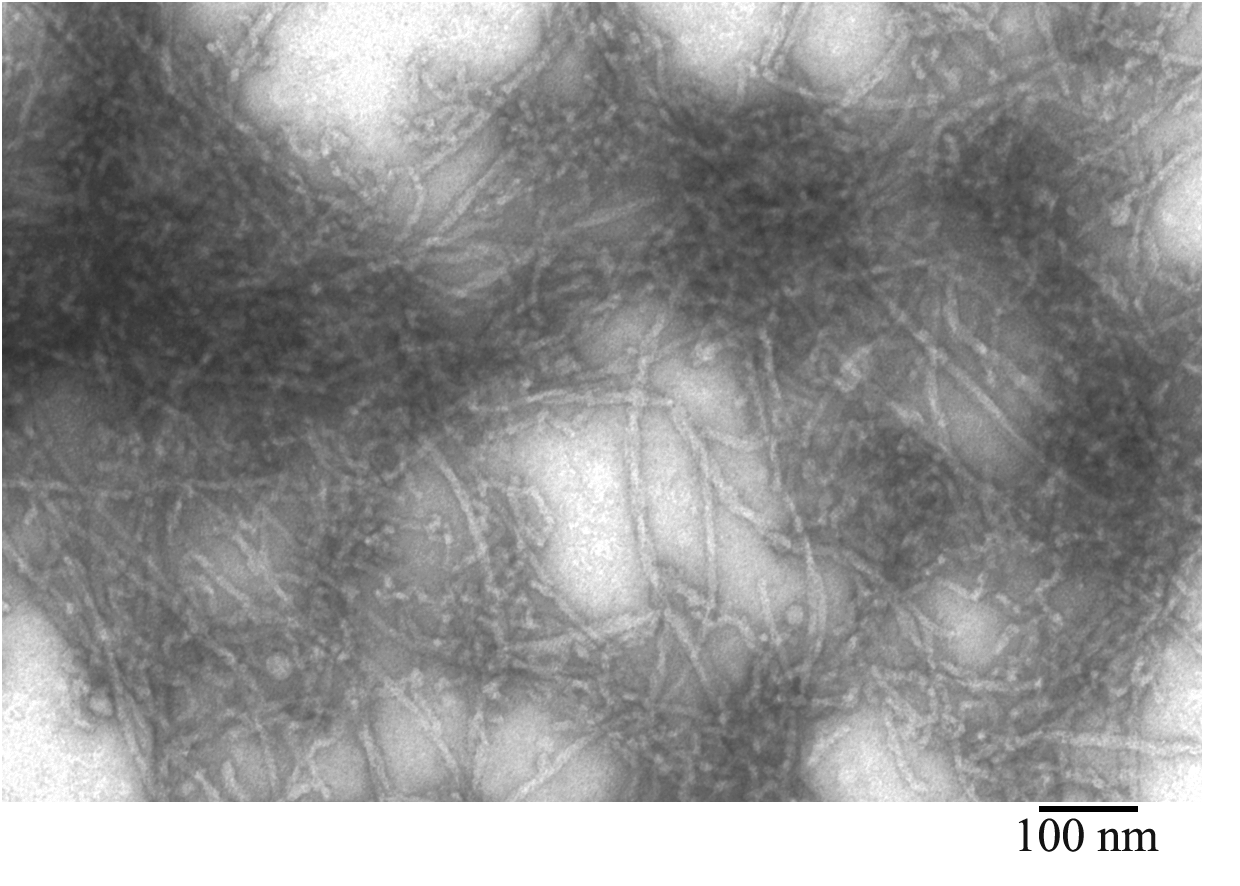

Supplement: Supplementary file 3 — Supplementary material [file mmc3.zip › SinAb42_24 h_Sup.tif]

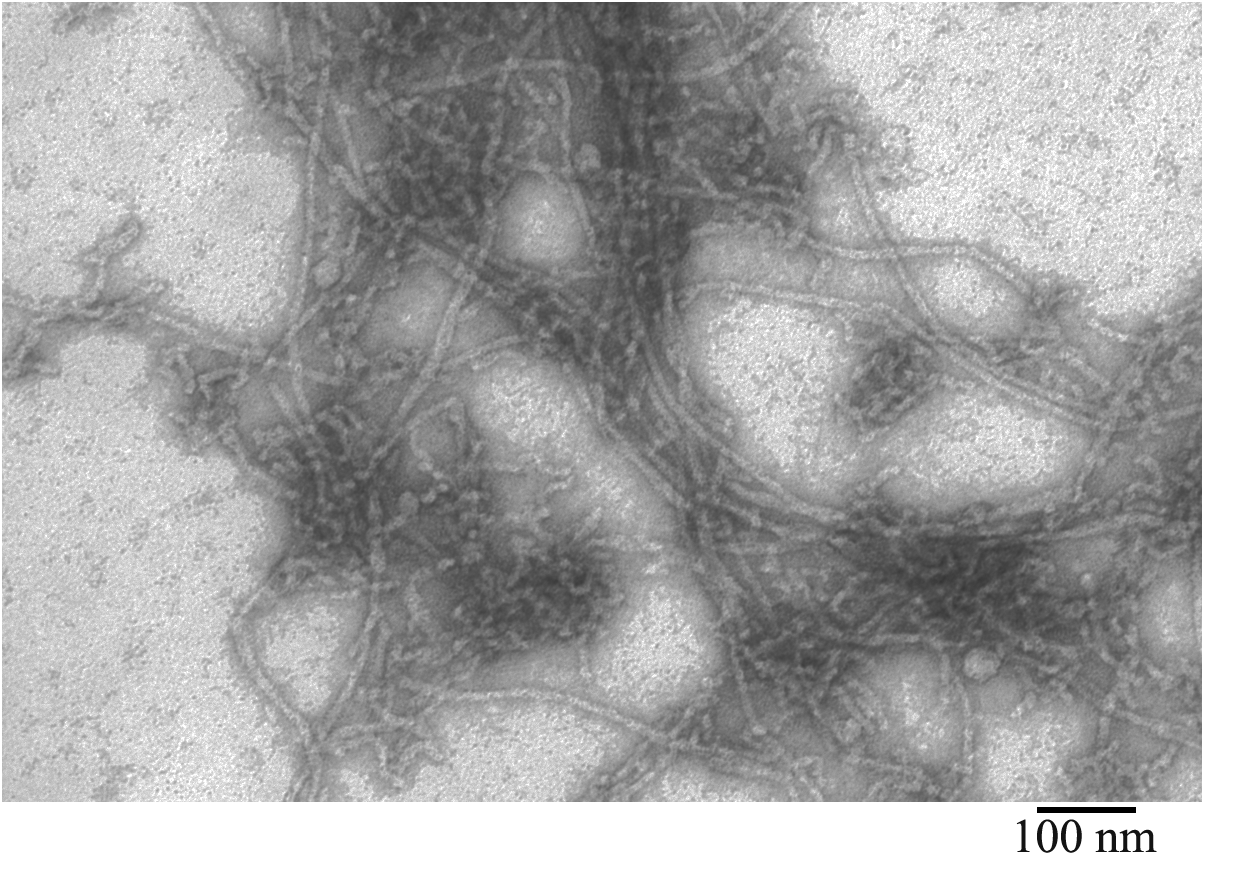

Supplement: Supplementary file 3 — Supplementary material [file mmc3.zip › SinAb42_8 h_Sup.tif]

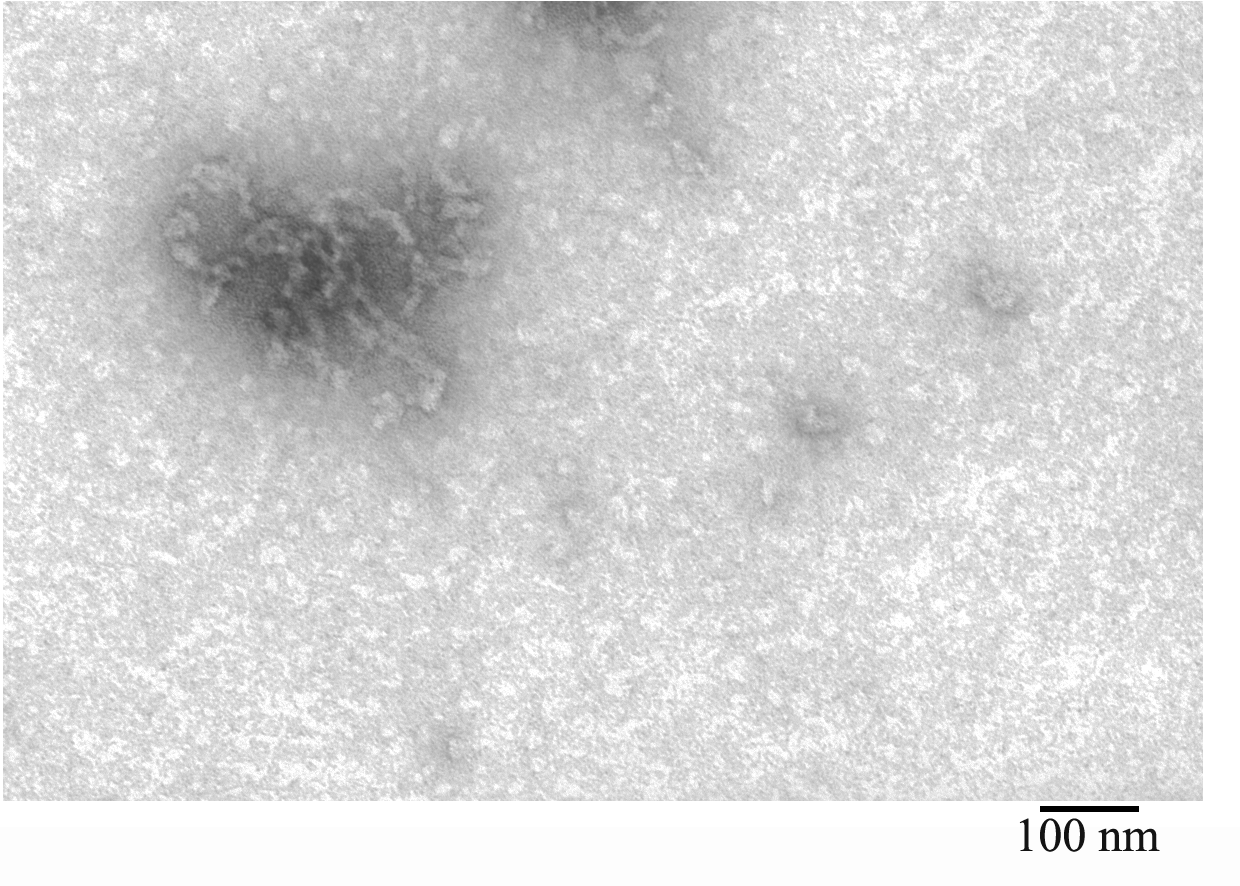

Supplement: Supplementary file 3 — Supplementary material [file mmc3.zip › SinAb42_0 h_Sup.tif]
